# Rice aboveground biomass estimation based on three-dimensional dry matter distribution integration model

**DOI:** 10.3389/fpls.2026.1924408

**Published:** 2026-08-27

**Authors:** Dandan Liu, Junjie Li, Min He, Yihan Wang, Jiahao Chen, Zhaoyang Pan, Teng Long, Yubin Lan, Zhenjiang Xu, Hong Liu, Yongbing Long

**Affiliations:** 1 College of Artificial Intelligence and Low-Altitude Technology, South China Agricultural University, Guangzhou, China; 2 National Center for International Collaboration Research on Precision Agricultural Aviationdc Pesticides Spraying Technology, South China Agricultural University, Guangzhou, China; 3 Guangdong Engineering Technology Research Center of Smart Agriculture, South China Agricultural University, Guangzhou, China; 4 College of Agriculture, South China Agricultural University, Guangzhou, China

**Keywords:** 3D-DMI framework, dual-step feature selection, machine learning, multi-source UAV remote sensing, rice aboveground biomass (AGB)

## Abstract

Accurate quantification of rice aboveground biomass (AGB) is critical for crop monitoring but remains challenging due to the complex nonlinearity arising from the coupling of plant density, spatial structure, and internal dry matter distribution. To address the limitations of single-source remote sensing, this study proposed a Three-Dimensional Dry Matter Distribution Integration (3D-DMI) model, which establishes a physically interpretable framework decomposing AGB into dry matter density ( ρ), horizontal projection distribution (*S*), and vertical cumulative distribution ( $h_{d}$) components. Guided by this framework, a core subset of six features (Red_650, MTCI, G_correlation, R_correlation, LPI, and HPA0_99) was extracted from UAV-based multispectral, RGB, and LiDAR data using a dual-step feature selection approach combining Maximum Information Coefficient (MIC) and Distance Correlation (dCor). A Random Forest (RF) regression model was then developed to estimate AGB across the entire growth season. The results demonstrated that the 3D-DMI model achieved excellent performance with an *R*^2^ of 0.920, an RMSE of 0.184 kg/m², and an RPD of 3.544, significantly outperforming any single-sensor approach. Single-feature analysis revealed that while LiDAR-derived structural features provided the fundamental basis for biomass estimation, they encountered inherent saturation bottlenecks during late growth stages. Feature contribution analysis based on SHAP further quantified that LiDAR-derived features dominated the estimation process (68.5% contribution), providing the volumetric basis, whereas RGB textures (18.3%) and multispectral features (13.3%) provided indispensable supplements. Ultimately, this study established a robust, physically grounded computational paradigm for high-precision UAV-based rice biomass monitoring across the entire growth cycle.

## 1 Introduction

Rice is one of the most important staple crops in the world, providing the primary food source for nearly half of the global population (Zhang et al., 2026; Jefferson et al., 2026). Rice aboveground biomass (AGB) is a key indicator for characterizing canopy growth status and productivity, closely related to photosynthetic accumulation and yield formation (Yue et al., 2023; Yang et al., 2023). Although previous studies have demonstrated the potential of remote sensing approaches for biomass estimation (He et al., 2025; Yang et al., 2026), these studies mainly focused on establishing statistical relationships between remote sensing observations and biomass. Yue et al. (2023) further provided a theoretical perspective by interpreting biomass from spatial distribution, vertical structure, and dry matter density dimensions, which inspired the physical basis of the proposed 3D-DMI framework. These factors are strongly intercoupled across different growth stages, resulting in pronounced spatial and temporal complexity in biomass dynamic changes, making its accurate characterization a critical and challenging task for rice production research.

Traditional methods for acquiring rice biomass rely mainly on destructive field sampling, which is labor-intensive and difficult to apply to large-scale monitoring (Derraz et al., 2023; Tong et al., 2019). With the rapid development of unmanned aerial vehicle (UAV) remote sensing, multispectral, RGB, and Light detection and ranging (LiDAR) sensors have been widely applied to capture crop phenotypic information with relatively high precision (Dai et al., 2026; Ji et al., 2026). For instance, RGB imagery has been extensively utilized to extract canopy cover and visual features in rice; notably, Nakajima et al. (2025) utilized purely UAV-based RGB imagery to estimate rice AGB from the transplanting to heading stages, achieving robust cross-regional estimation accuracy (adjusted*R*^2^> 0.80) across multiple independent locations. Similarly, Li et al. (2025a) utilized UAV-based multispectral imagery to extract vegetation indices and wavelet textures for estimating rice biomass, achieving accuracy (*R*^2^ = 0.782) during the pre-heading stages, while the accuracy decreased to 0.769 during the post-heading stages and further declined to 0.732 across the entire growth period. Furthermore, Revenga et al. (2022) utilized UAV-LiDAR to derive structural metrics for barley and winter wheat, achieving robust biomass estimation across the entire growing season with *R*^2^ values reaching 0.93 and 0.89, respectively, thereby effectively overcoming the saturation issues common in optical remote sensing.

While these studies successfully demonstrate the immense potential of individual sensors in extracting specific canopy traits, relying solely on single-source remote sensing data reveals inherent physical bottlenecks when estimating AGB across the full reproductive cycle of rice.

First, Multispectral Vegetation Indices (VIs) are susceptible to background noise, saturation, and phenological instability (Tilling et al., 2007). For instance, in a comprehensive multi-sensor study on rice, Yang et al. (2023) explicitly evaluated the independent performance of various VIs and found that they rapidly saturate upon canopy closure and experience significant hysteresis after panicle emergence, resulting in inadequate independent estimation accuracy (*R*^2^ < 0.5). This limitation is further supported by Niu et al. (2025), who noted in maize monitoring that estimation accuracy was hindered by background soil noise during early growth and spectral saturation during high-biomass stages (*R*^2^ ranged from 0.51 to 0.68). Additionally, Liu et al. (2023a) found in potato estimation that optical spectral features lost sensitivity over the entire growth period due to changing canopy composition, yielding lower accuracy (*R*^2^ < 0.61) compared to structural features.

Second, both textural and structural features derived from RGB imagery are limited by passive sensing characteristics. Yue et al. (2019) found that the predictive performance of texture metrics was heavily dependent on ground resolution and failed to characterize high-biomass wheat. Furthermore, regarding structural extraction, Maimaitijiang et al. (2020) compared LiDAR and RGB photogrammetry in sorghum monitoring and revealed that RGB-based point clouds suffer from limited canopy penetration, leading to significant underestimation of canopy height and Leaf Area Index in dense canopies compared to active LiDAR sensors.

Third, although LiDAR sensors excel at characterizing canopy geometry, they lack the spectral sensitivity to detect internal physiological status and dry matter density (Hosoi and Omasa, 2012, 2009). Revenga et al. (2022) noted in barley monitoring that LiDAR intensity metrics did not correlate consistently with biomass, failing to provide sufficiently descriptive physiological features. Moreover, Maimaitijiang et al. (2019) demonstrated in soybean phenotyping that structural metrics alone were insufficient for robust estimation, as plants with identical volumes often possessed different biomass weights due to variations in internal biochemical content, necessitating the inclusion of spectral indices to represent bulk density.

These limitations highlight that a single remote sensing information source is inherently insufficient to accurately characterize rice AGB throughout the entire growing season. The physical accumulation of AGB is a complex three-dimensional process encompassing horizontal canopy expansion, vertical structural growth, and internal dry matter accumulation. Because an individual sensor typically captures only partial spectral or structural information, it fails to comprehensively represent these complementary multidimensional traits, inevitably leading to estimation bottlenecks (Jin et al., 2025; Zhang et al., 2025; Kong et al., 2025).

Multi-source remote sensing data fusion provides a promising pathway for addressing these limitations by combining complementary information (Wang et al., 2024; Lin et al., 2023; Zhang et al., 2024). Recent studies have demonstrated the efficacy of integrating spectral and structural features. For example, Dai et al. (2024) developed a multidimensional rice AGB estimation model by combining multispectral VIs with spatial parameters (plant height and canopy cover) and phenological stages, achieving high accuracy (*R*^2^ = 0.88) specifically during the mid-tillering to flowering stages. Similarly, Li et al. (2025) integrated high-frequency textures and color features from RGB images with vegetation indices from multispectral images, effectively mitigating the spectral saturation problem and achieving highly accurate estimations of rice aboveground biomass across the tillering, jointing, and heading stages (*R*^2^ = 0.8564).

However, many existing studies treat fusion as a statistical concatenation of features by simply stacking spectral, textural, and structural variables into machine learning models (Eleyan, 2023; Zeng et al., 2022; Naqvi et al., 2024), lacking a unified theoretical framework to explicitly link remote sensing variables to the physical components of biomass formation. There is a critical need to treat AGB estimation (Marquart et al., 2025; Liu et al., 2023b; Baig et al., 2017) as a three-dimensional dry matter distribution modeling problem and to identify specific remote sensing proxies that can effectively characterize the governing physical variables (Anees et al., 2024). These limitations indicate that existing approaches still lack a unified physical framework that explicitly links remote sensing observations with the three-dimensional components governing crop biomass accumulation.

**Figure 1:**
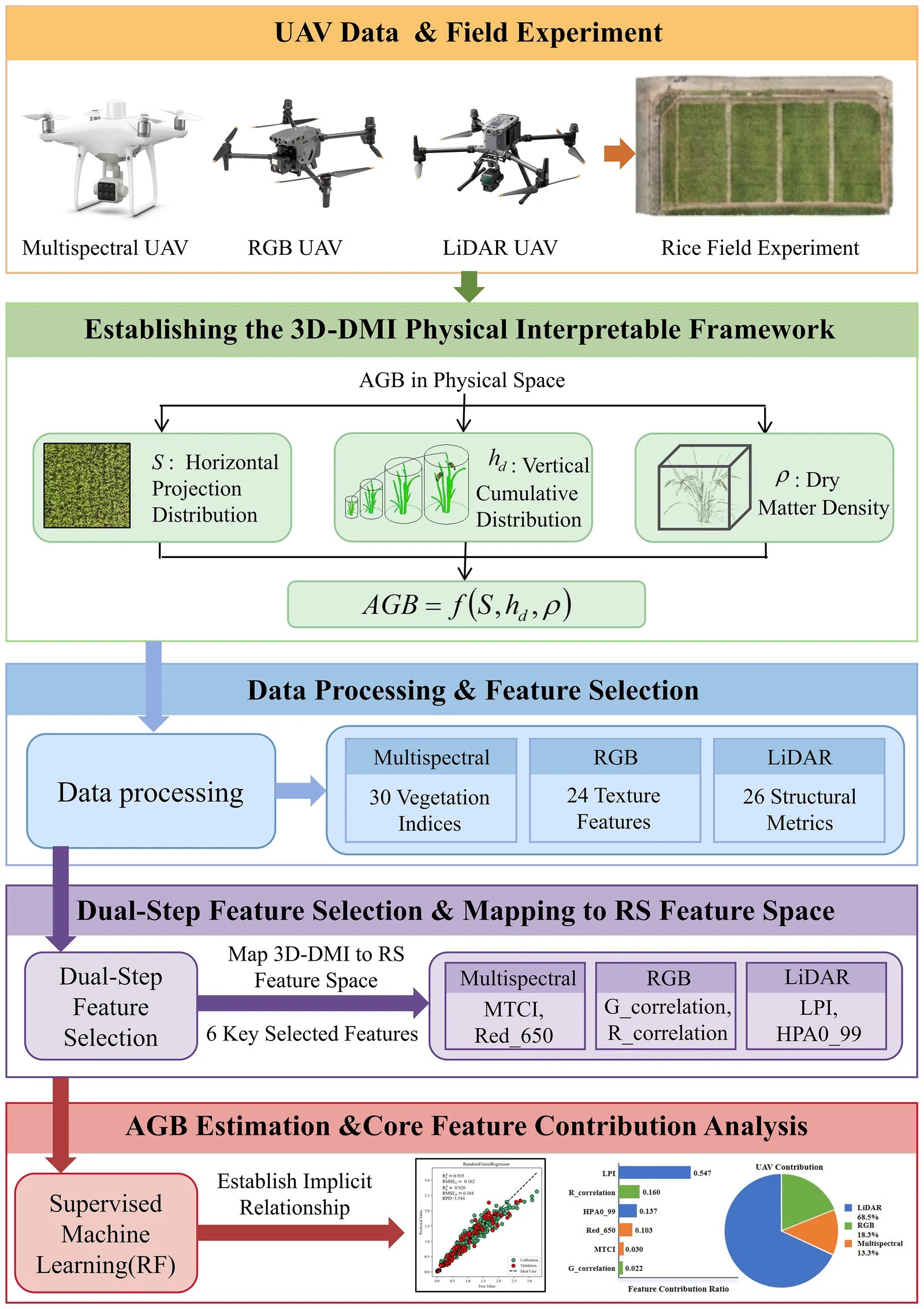
Flow chart of the experiment.

This issue was addressed in the present study by proposing a three-dimensional dry matter distribution integration (3D-DMI) model to establish a physically interpretable guiding framework for AGB estimation across the entire growth stage. Firstly, 3D-DMI model was established to describe the AGB in physical space using three dimensions, namely the horizontal projection distribution component of the rice canopy (*S*), the vertical cumulative distribution component ( $h_{d}$), and the dry matter density component (ρ). A dual-step feature selection approach was then employed to map the 3D-DMI model from physical space into UAV remoting sensing feature space, with six key features systematically extracted from RGB, multispectral, and LiDAR data. Finally, 3D-DMI model was solved by supervised machine learning to establish the implicit expressive relationship relation between the rice AGB and six selected key features and estimation model with high accuracy was established to predict rice AGB across the full growth stage. Furthermore, we conducted a feature contribution analysis based on SHAP (Shapley Additive exPlanations) to quantify the potential contributions of the horizontal projection distribution component, the vertical cumulative distribution component, and the dry matter density component in the estimation process. The experimental workflow is presented in **Figure 1**.

## 2 Materials and methods

### 2.1 Study area and experimental design

**Figure 2:**
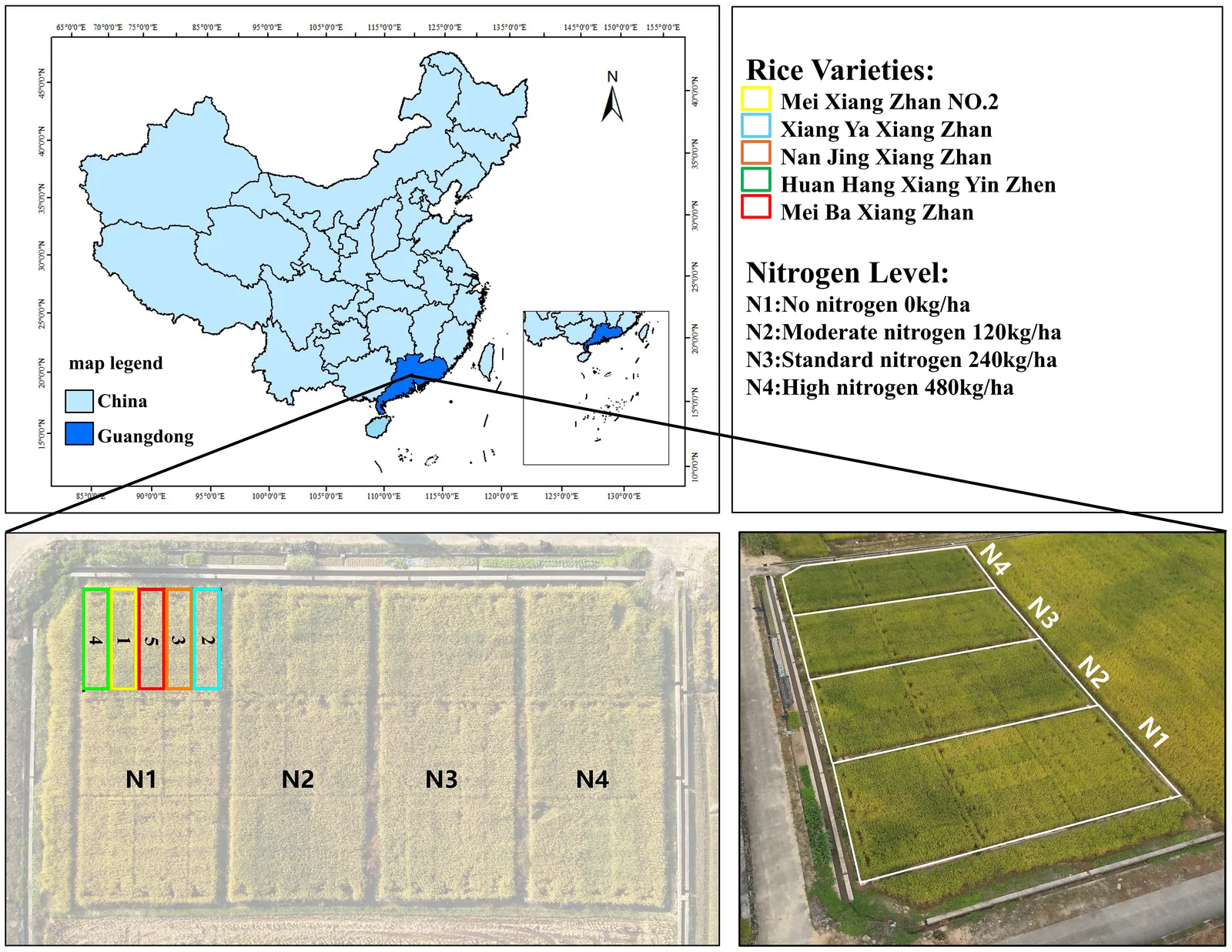
Overview of the study area and experimental design.

A field experiment was conducted from September to October 2024 at Ningxi Experiment Base in Zengcheng, South China Agricultural University, Guangzhou, China (37°410 N, 116°370 E). The region features a subtropical monsoon climate with an average annual temperature of approximately 21.8 °C and an annual precipitation of 1800–2000 mm. The soil has an organic matter content of 1.8–2.2%, representing typical rice cultivation conditions in South China. An overview of the study area and the specific experimental layout is shown in **Figure 2**.

During the experiment, five common rice varieties were cultivated: V1 (Mei Xiang Zhan No.2), V2 (Xiang Ya Xiang Zhan), V3 (Nan Jing Xiang Zhan), V4 (Hua Hang Xiang Yin Zhen), and V5 (Mei Ba Xiang Zhan). For each rice variety, four nitrogen application levels were established: N1 (0 kg/ha), N2 (120 kg/ha), N3 (240 kg/ha), and N4 (480 kg/ha). Each treatment was replicated three times, resulting in a total of 60 plots, which were separated by ridges and vertical polyethylene water barriers to prevent lateral nutrient and water seepage. Within each plot, rice was planted with a row spacing of 25 cm × 16 cm and a planting density of 25 plants per square meter, ensuring uniformity and standardization across the experiment.

### 2.2 Ground-based rice biomass data acquisition

Destructive sampling was conducted 10 times at key agronomic growth stages throughout the growing season, spanning from the early tillering stage (September 5) to maturity (November 29). At each sampling event, three hills of rice plants were randomly selected from each plot. The sampled plants were separated into their constituent organs (leaves, stems, and panicles), heat-treated at 105 °C for enzyme deactivation, and subsequently dried at 80 °C to a constant weight. Rice AGB ( $kg/m^{2}$) was calculated using Equation 1:

(1) $$AGB=\frac{D\times (W_{1}+W_{2}+W_{3})}{3}$$

where AGB is the aboveground biomass per unit area; *D* represents the planting density (hills/m^2^); and *W_1_*, *W_2_*, and *W_3_* represent the total dry weights of the leaves, stems, and panicles from the three sampled hills, respectively. A total of 598 valid samples were obtained after outlier removal.

### 2.3 Data acquisition and processing

To construct a rice biomass estimation model based on multi-source UAV remote sensing features, multiple UAV platforms were employed to acquire multispectral, RGB and LIDAR data of the rice canopy. A Phantom 4 UAV equipped with a multispectral camera was deployed to capture multispectral information. The camera comprised one visible sensor and five multispectral bands: 450 nm (blue), 560 nm (green), 650 nm (red), 730 nm (red-edge), and 840 nm (near-infrared). A standard reflectance panel was used for radiometric calibration prior to each flight to ensure the accuracy of reflectance measurements. The M30T UAV carrying a twelve-megapixel wide-angle camera was used to obtain high-resolution RGB images, and the M350T UAV carrying the Zenmuse L2 LiDAR system was used to obtain three-dimensional canopy point cloud data. All UAV missions were performed with consistent flight parameters with an altitude of 12 m and a speed of 2 m per second, and both forward and side overlaps of 75 percent, ensuring complete coverage and adequate overlap of the study area.

During data processing, the raw multispectral and RGB images were processed in DJI Terra for image reconstruction, geometric correction, and radiometric calibration. The generated images were then clipped according to predefined plot boundaries to extract the mean single-band reflectance values and RGB data, with reflectance values normalized to mitigate illumination differences. For the LiDAR data, raw point clouds acquired by the Zenmuse L2 sensor were first reconstructed into LAS files using DJI Terra. Subsequently, point cloud denoising and elevation normalization based on ground point classification were performed. Finally, the normalized data were clipped according to each plot’s coordinates to obtain the point cloud datasets for subsequent spatial structural feature extraction.

### 2.4 3D-DMI model in physical space

Rice AGB refers to the cumulative dry matter of all aboveground organs per unit ground area. From the perspective of canopy architecture and physiological development, biomass accumulation is fundamentally a process of three-dimensional spatial distribution and internal matter filling. More specifically, AGB can be calculated as a sum of the dry matter in the space occupied by the rice canopy.

Before constructing the 3D-DMI framework, we systematically reviewed previous studies on remote sensing-based crop biomass estimation and found that most existing studies mainly focused on one or two dimensions of biomass composition. For example, many studies analyzed canopy horizontal distribution characteristics, such as leaf area index and spectral indices, or relied mainly on vertical structural information, such as plant height and canopy surface models. However, few studies integrated horizontal distribution, vertical structure, and internal dry matter accumulation into a unified physical framework.

Among these studies, the work of Yue et al. (2023) provided direct theoretical inspiration and methodological reference for this study. Their model decomposed crop aboveground biomass into leaf biomass and biomass of vertically growing organs, including stems and reproductive organs. In this model, plant distribution per unit area reflected horizontal canopy distribution and density characteristics, plant height represented vertical spatial structure, and dry matter density was defined as the average dry matter weight of leaves, stems, and reproductive organs per unit volume. Inspired by this concept, we further considered that rice aboveground biomass is physically associated with three relatively independent and complementary factors: horizontal spatial distribution, vertical spatial structure, and internal dry matter density. These three components provide the theoretical basis for the construction of the 3D-DMI framework.

The 3D-DMI model is then used to formulate rice AGB as the volume integration of dry matter mass within the canopy space, as given in Equation (2):

(2) $$AGB=\int_{0}^{C_{h}}S(x,y)\rho (x,y,z)dxdydz$$

Here, S(x,y) represents the horizontal projection distribution of the rice canopy, reflecting the lateral occupancy of plant organs; ρ(x,y,z) represents the spatial distribution of dry matter density, reflecting the local mass concentration of all aboveground organs at the 3D coordinate (x,y,z); and $C_{h}$ denotes the canopy height.

**Figure 3:**
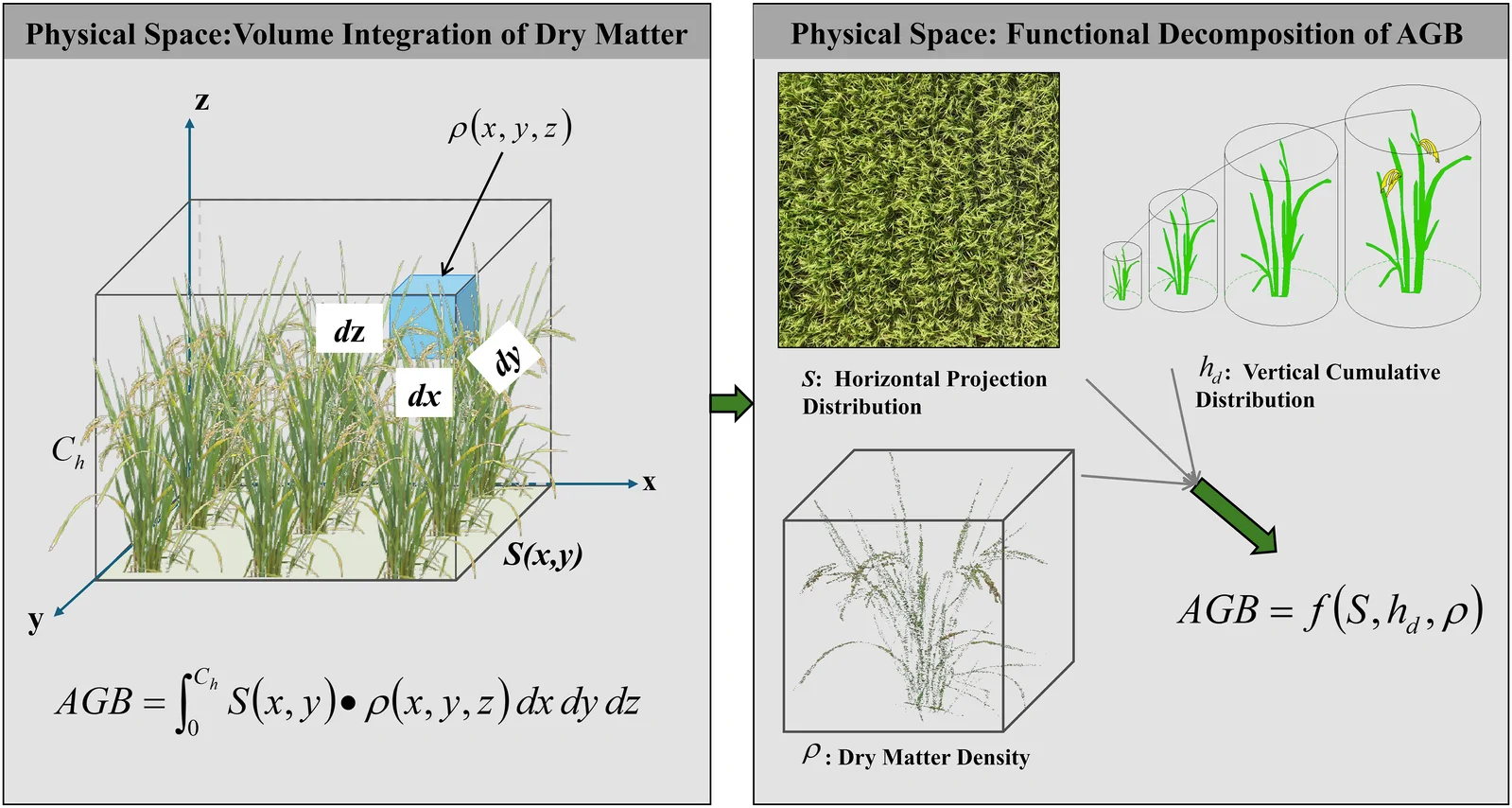
Mathematical formulation of the 3D-DMI model.

The 3D-DMI model can be understood as illustrated in **Figure 3**, which decomposes the model into three functional components in physical space: the horizontal projection distribution *S*, the vertical cumulative distribution $h_{d}$, and the dry matter density ρ. Thus, AGB can be expressed as a generalized nonlinear function, as given in Equation (3):

(3) $$AGB=f(S,h_{d},\rho )$$

This formulation establishes a physically interpretable framework for estimating rice AGB and also guides the subsequent feature selection for mapping the model from physical space to remote sensing feature space. Specifically, the horizontal component *S* corresponds to lateral canopy coverage and spatial arrangement patterns, necessitating remote sensing features that effectively describe the two-dimensional horizontal distribution. The vertical component $h_{d}$ reflects the vertical biomass profile and volume accumulation, requiring metrics that quantify the 3D canopy architecture and height cumulative characteristics. Finally, the dry matter density component ρ  characterizes internal tissue compactness and biochemical status, calling for features sensitive to physiological vigor and matter concentration.

From a physiological perspective, the three components of the 3D-DMI model represent distinct but complementary processes underlying biomass accumulation. The horizontal projection distribution describes canopy spatial occupancy, which determines the interception and utilization efficiency of solar radiation through leaf arrangement, overlap, and canopy gaps. The vertical cumulative distribution represents the development of plant height, internode elongation, tillering expansion, and vertical leaf stratification, which together define the three-dimensional canopy architecture. The dry matter density reflects the accumulation and allocation of photosynthetic assimilates within aboveground organs, representing the process of dry matter production and storage during crop development.

### 2.5 Construction of UAV remote sensing feature space

#### 2.5.1 Construction of the feature pool for selection

To map the physical variables of the 3D-DMI model into a remote sensing context, a comprehensive feature pool was initially established. A total of 80 features were extracted from UAV-derived multispectral, RGB, and LiDAR data to independently characterize the dry matter density ( ρ), horizontal projection distribution (*S*), and vertical cumulative distribution ( $h_{d}$) of the rice canopy.

First, for the multispectral data, we extracted mean reflectance values from five spectral bands and calculated 30 vegetation indices. Optical spectral responses have long been proven to correlate highly with crop photosynthetic capacity and total dry-matter accumulation (Tucker et al., 1981). Furthermore, narrow-band indices are specifically sensitive to biochemical concentrations per unit mass and area (Blackburn, 1998). Therefore, these VIs serve as ideal proxies for the dry matter density. To address potential saturation issues under high biomass conditions, we incorporated red-edge and structure-sensitive indices such as TNDVI, NDRE, MTCARI, and the MERIS Terrestrial Chlorophyll Index (MTCI), which maintains high sensitivity at elevated chlorophyll levels (Duan et al., 2019; Dash and Curran, 2004).

Second, for the RGB images, the Gray Level Co-occurrence Matrix (GLCM) algorithm (Haralick et al., 1973) was utilized to quantify the spatial heterogeneity of the canopy. GLCMs were computed across the three visible channels (Red, Green, Blue) at a pixel distance of 1. To ensure rotational invariance, textures were calculated in four main directions (0°, 45°, 90°, 135°) and averaged. Eight texture metrics (Mean, Variance, Homogeneity, Contrast, Dissimilarity, Entropy, ASM, and Correlation) were derived per channel, yielding 24 texture features. These features effectively capture canopy roughness and horizontal spatial arrangement, making them highly correlated with the horizontal projection distribution.

Third, LiDAR sensors, which actively emit laser pulses to penetrate canopy gaps, were employed to characterize the 3D structural profile. After denoising and normalization, 26 structural metrics were extracted from the point clouds. Key metrics included the Laser Penetration Index (LPI), defined as the ratio of ground returns to total returns. LPI characterizes the canopy gap fraction (Luo et al., 2024b), directly reflecting the horizontal projection distribution. Additionally, height percentiles (e.g., H99, H95, H_mean) and Height Percentile Area (HPA) metrics were adopted to strictly quantify the vertical cumulative distribution.

The above procedure established a multi-source feature pool containing 80 variables, which comprised 30 multispectral vegetation indices, 24 RGB texture features, and 26 LiDAR structural metrics. The feature pool provided a robust basis for the subsequent feature selection to identify the most representative remote sensing features most closely the physical variables ( ρ, *S*, $h_{d}$).

#### 2.5.2 Feature selection for UAV remote sensing feature space

A dual-step feature selection approach combining the Maximum Information Coefficient (MIC) and Distance Correlation (dCor) was employed to identify the features most strongly associated with the three physical components. First, MIC values were calculated for all features within each sensor category. As a robust measure, MIC is highly capable of detecting both linear and non-linear functional relationships (Reshef et al., 2018). Based on these values, the top 10 features demonstrating the strongest associations with AGB in each category were retained.

Subsequently, to differentiate features with highly similar MIC values and further distinguish the statistical dependence of the candidates, dCor was introduced to re-rank these top 10 features. Unlike MIC, dCor provides a highly continuous measure of dependence, enabling precise differentiation. Based on the dCor ranking, the top 2 features from each sensor category were finalized as model inputs. As a result, a core subset of 6 key features (2 multispectral, 2 RGB, and 2 LiDAR) was identified, serving as the optimal remote sensing proxies for the physical variables in the 3D-DMI model.

### 2.6 3D-DMI modelling in UAV remote sensing feature space

Following feature selection, nine supervised machine learning (Cheng et al., 2025; Chen et al., 2025; Li et al., 2025b; Jiang et al., 2023) algorithms were evaluated to establish the implicit expressive relationship relation between rice AGB and six selected key features, aiming to determine the optimal estimator for the 3D-DMI model. These algorithms included Support Vector Regression (SVR), K-Nearest Neighbors (KNN), Random Forest (RF), Gradient Boosting Regression (GBR), AdaBoost, Bagging, Extra Trees, XGBoost, and Kernel Ridge Regression (KRR). The modeling workflow integrated hyperparameter optimization, model calibration, and quantitative evaluation. During training, the Optuna automated framework combined with 5-fold cross-validation (5-fold CV) was utilized to search for optimal hyperparameter combinations through 100 iterations. Subsequently, the optimal hyperparameters were used to retrain the final model on the entire training set, which was then applied to the independent testing set. The model accuracy was quantified using three complementary metrics of Coefficient of Determination (*R*^2^), Root Mean Square Error (RMSE), and Residual Prediction Deviation (RPD). The calculation formulas as given in (Equations 4–6):

(4) $${\text{R}}^{2}=1-\frac{\sum_{i=1}^{n}{\left(y_{i}-{\hat{y}}_{i}\right)}^{2}}{\sum_{i=1}^{n}{\left(y_{i}-\bar{y}\right)}^{2}}$$

(5) $$\text{RMSE}=\sqrt{\frac{1}{n}\sum_{i=1}^{n}{\left(y_{i}-{\hat{y}}_{i}\right)}^{2}}$$

(6) $$\text{RPD}=\frac{SD}{RMSE}$$

where $y_{i}$ and ${\hat{y}}_{i}$ represent the measured and predicted AGB values of the i−th sample, respectively; $\bar{y}$ is the mean of the measured values; *n* is the total number of samples; and SD is the standard deviation of the measured values. *R*^2^ measures the fitting degree; RMSE reflects the prediction deviation; and RPD indicates the generalization ability.

## 3 Result

### 3.1 Statistical analysis of rice AGB data

**Figure 4:**
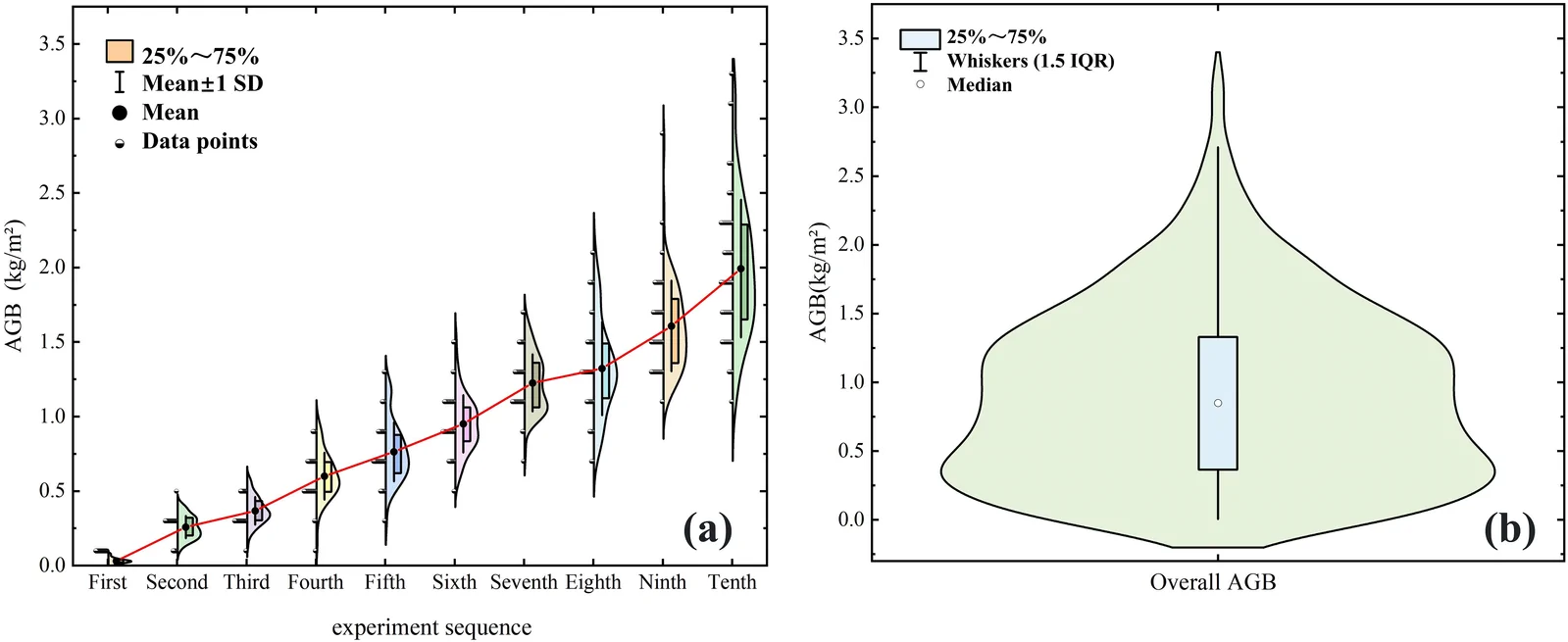
**(a)** Half-violin plots of rice AGB at different growth stages over the whole growth period; **(b)** Overall violin plot of AGB distribution.

**Figure 4A** visualizes the dynamic changes in rice AGB across different growth stages using violin plots, while **Figure 4B** presents the distribution of all rice AGB data. Over the entire growth cycle, the rice AGB ranged from 0.004 to 3.24 kg/m², a range that fully characterizes continuous biomass accumulation process from the seedling to maturity stage. The mean AGB at the seedling stage was relatively low (approximately 0.03 kg/m²), increased sharply during tillering stage, and continued to rise in subsequent stages, with rapid biomass accumulation observed during gain filling and maturity stages. AGB reached its maximum during maturity stage, with the mean value exceeding 2 kg/m².

In summary, the constructed dataset encompasses the complete rice growth cycle from tillering to maturity. By capturing both a wide range of biomass values and critical temporal dynamics, it provides a diverse and reliable foundation for subsequent feature analysis and AGB estimation modeling.

### 3.2 Core feature selection for 3D-DMI characterization

**Figure 5:**
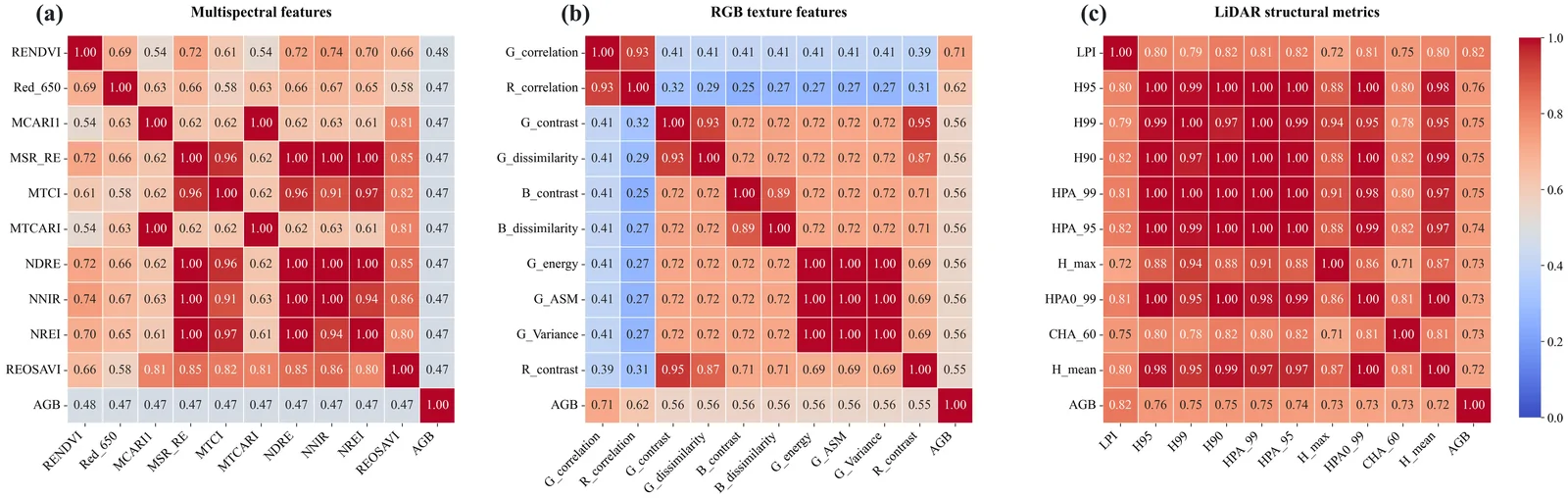
MIC heatmaps of candidate features with rice AGB. **(a)** Multispectral features; **(b)** RGB texture features; **(c)** LiDAR structural metrics.

**Figure 6:**
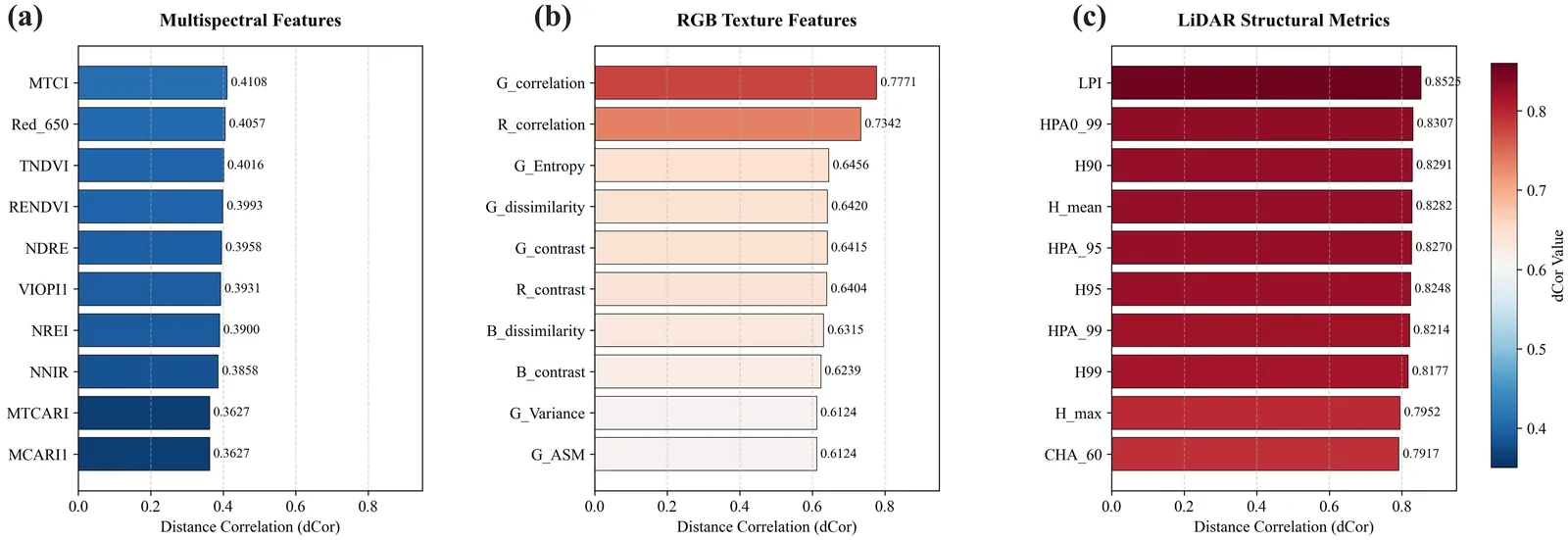
dCor ranking of candidate features. **(a)** Multispectral features; **(b)** RGB texture features; **(c)** LiDAR structural metrics.

To extract effective proxies from the high-dimensional UAV remote sensing feature pool for characterizing the 3D-DMI model, feature screening was conducted using the dual-step feature selection approach. MIC was first employed to screen the features with significant nonlinear correlations to rice AGB and the top 10 features for each sensor category were visualized in the heatmaps of **Figure 5**. Although MIC effectively identified features with strong associations, the top 10 features in **Figure 5A** and the nine second-level important features in **Figure 5C** showed no significant difference, making their priority difficult to distinguish rigorously. Therefore, dCor analysis was further applied to the top 10 features selected by MIC to rigorously rank their statistical dependence, with detailed results presented in **Figure 6**. Following this dual-step selection procedure, the top two features from each sensor category were selected as the core feature subset. The definitions and calculation methods of these six core features are listed in **Table 1**.

**Table 1:** Variables derived from multi-source UAV data used in this study.

Sensor	Variable name	Symbol	Definition/formula	Physical component in 3D-DMI	Reference
Multispectral	MERIS Terrestrial Chlorophyll Index	MTCI	$\frac{R_{NIR}-R_{REG}}{R_{REG}-R_{RED}}$	Dry Matter Density (*ρ*)	Dash and Curran, 2004
	Red Reflectance	Red_650	Mean reflectance at 650 nm	Dry Matter Density (*ρ*)	Blackburn, 1998
RGB	Green Band Correlation	G_correlation	GLCM Correlation (Green band)	Horizontal Projection Distribution (*S*)	Haralick, 1973
	Red Band Correlation	R_correlation	GLCM Correlation (Red band)	Horizontal Projection Distribution (*S*)	Haralick, 1973
LiDAR	Laser Penetration Index	LPI	$\frac{N_{ground}}{N_{total}}$	Horizontal Projection Distribution (*S*)	Luo et al., 2024b
	Height Profile Area Integral	HPA0_99	$\int_{H_{0}}^{H_{99}}f(z)\;dz$	Vertical Cumulative Distribution (*h_d_*)	Luo et al., 2024b

The quantitative analysis presented in **Figure 6** indicates that specific features from each sensor type exhibited dominant correlations with AGB, effectively representing different physical components of the 3D-DMI model. Regarding the multispectral features (**Figure 6A**), MTCI and Red_650 achieved the highest distance correlation values of 0.4108 and 0.4057, respectively. These features were identified as key proxies for the dry matter density. Specifically, the Red_650 band is situated between the primary absorption peaks of chlorophyll a (680 nm) and chlorophyll b (635 nm), as identified by Blackburn (1998), enabling it to capture the combined strong absorption effects of total pigments. Furthermore, Dash and Curran (2004) established that MTCI remains sensitive even at high chlorophyll levels, effectively mitigating the saturation issues common in dense canopies. This characteristic ensures robust sensitivity for biomass estimation in cereal crops such as rice (Wang et al., 2022).

Although Red_650 is sensitive to canopy pigment characteristics, pigment status is closely associated with crop physiological activity, nitrogen-related status, and dry matter accumulation during rice growth. The temporal variation of chlorophyll content and nitrogen status reflects changes in photosynthetic capacity and assimilate production, which directly contribute to biomass accumulation and dry matter allocation. Therefore, the red reflectance at 650nm provides indirect information on the physiological processes associated with dry matter accumulation and can serve as an effective spectral proxy for the dry matter density component in the 3D-DMI framework.

In terms of the RGB features shown in **Figure 6B**, the texture metrics G_correlation and R_correlation demonstrated markedly stronger associations, with dCor values reaching 0.7771 and 0.7342, respectively. These metrics contribute to characterizing the horizontal projection distribution. Specifically, texture correlation quantifies the periodicity and uniformity of gray levels in the image plane, Furthermore, within the LiDAR structural metrics presented in **Figure 6C**, the LPI and the HPA0_99 imposed the strongest statistical dependence on AGB, achieving correlation values of 0.8525 and 0.8307, respectively. LPI serves as a critical proxy for the horizontal projection distribution alongside RGB textures, as it reflects horizontal canopy porosity by calculating the ratio of ground returns. Meanwhile, HPA0_99 serves as the definitive proxy for the vertical cumulative distribution, as it mathematically integrates the height profile to describe the accumulated volume of the canopy skeleton. The results show that the correlation of LiDAR features is significantly higher than that of RGB and multispectral features, which is consistent with the premise that rice AGB is a volumetric entity. Consequently, the six top-ranked features identified through the dual-step feature selection approach were finalized as the core subset for subsequent modeling.

### 3.3 3D-DMI characterization and analysis

#### 3.3.1 Algorithm evaluation and optimal solver selection

The 3D-DMI model describes the complex non-linear relationship between rice AGB and the six selected UAV remote sensing features. Since this relationship cannot be solved analytically, we adopted supervised machine learning algorithms to capture the implicit mapping (Matiza et al., 2024; Dhakal et al., 2023; Luo et al., 2024a). To identify the optimal solver, nine regression models were evaluated: SVR, KNN, RF, GBR, AdaBoost, Bagging, Extra Trees, XGBoost, and KRR.

The comparative performance is detailed in **Table 2**. The results demonstrated that the RF-based 3D-DMI model achieved the best performance, with a *R*^2^ of 0.920, RMSE of 0.184 kg/m², and an RPD of 3.544. This superior performance is attributable to the ensemble nature of RF. As noted by Belgiu and Drăguţ (2016), the RF model is highly effective in handling the high-dimensional data and multicollinearity common in remote sensing. Furthermore, its bagging mechanism significantly mitigates overfitting compared to single decision trees, making it well-suited for modeling the complex coupling between UAV features and rice AGB across the whole growth period.

**Table 2:** Performance comparison of nine machine learning algorithms for rice AGB estimation.

Regression modeling algorithm	Training set		Test set		RPD
	R^2^_C_	RMSE_C_	R^2^_V_	RMSE_V_	
RF	0.935	0.162	0.920	0.184	3.544
SVR	0.850	0.245	0.873	0.232	2.816
KNN	0.854	0.242	0.895	0.210	3.105
GBR	0.896	0.204	0.895	0.211	3.099
AdaBoost	0.878	0.221	0.855	0.247	2.641
Bagging	0.977	0.096	0.907	0.198	3.292
Extra Trees	0.775	0.300	0.699	0.356	1.833
XGBoost	0.800	0.284	0.823	0.273	2.387
KRR	0.832	0.260	0.848	0.253	2.577

#### 3.3.2 Performance analysis of multi-sensor information fusion

**Figure 7:**
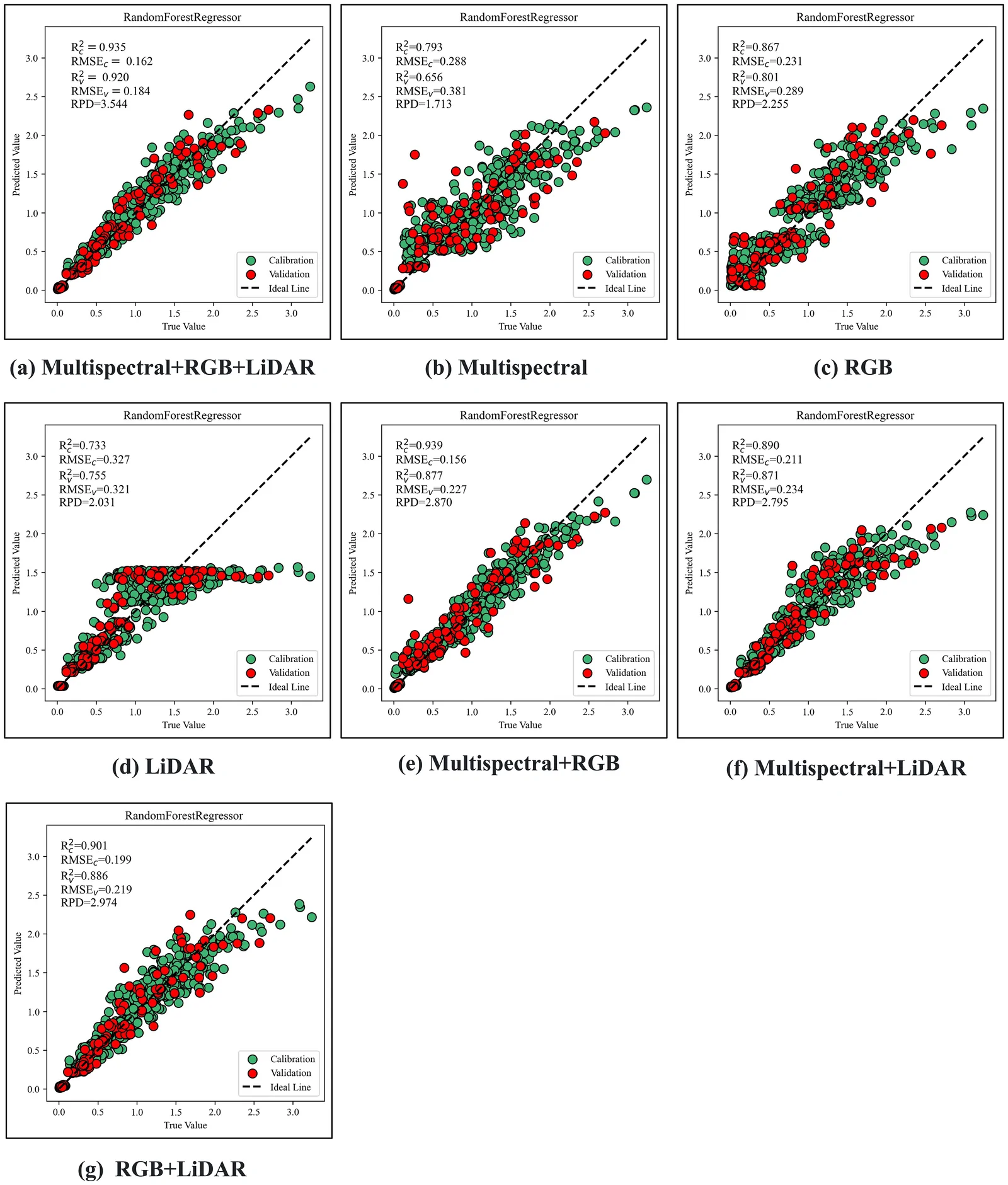
Scatter plots of measured versus predicted rice AGB under different sensor feature combinations. **(a)** Multispectral + RGB + LiDAR; **(b)** Multispectral; **(c)** RGB; **(d)** LiDAR; **(e)** Multispectral + RGB; **(f)** Multispectral + LiDAR; **(g)** RGB + LiDAR.

To further understand the contribution of the three physical components, namely dry matter density ( ρ), horizontal projection distribution (*S*), and vertical cumulative distribution ( $h_{d}$), we compared the modeling performance of different sensor combinations using the RF algorithm (**Table 3**; **Figure 7**).

**Table 3:** Performance comparison of rice AGB estimation models under different sensor feature combinations using the RF.

Feature combination scenarios	Multispectral	RGB	LiDAR	Training set		Test set		RPD
				Rc^2^	RMSE_C_	R^2^_V_	RMSE_V_	
All Combined	✓	✓	✓	0.935	0.162	0.920	0.184	3.544
Single Sensor	✓	–	–	0.793	0.288	0.656	0.381	1.713
	–	✓	–	0.867	0.231	0.801	0.289	2.255
	–	–	✓	0.733	0.327	0.755	0.321	2.031
Dual Sensors	✓	✓	–	0.939	0.156	0.877	0.227	2.870
	✓	–	✓	0.890	0.211	0.871	0.234	2.795
	–	✓	✓	0.901	0.199	0.886	0.219	2.974

In the single-sensor scenarios, the results revealed significant performance disparities grounded in physical mechanisms. The model based solely on multispectral features representing dry matter density achieved the lowest accuracy (*R*^2^ = 0.656), which aligns with the scientific understanding that spectral indices primarily characterize biochemical concentration rather than volume. In contrast, models incorporating spatial structural features achieved significantly higher accuracies, with the RGB model (representing the horizontal projection distribution) and the LiDAR model (representing both vertical and horizontal distributions) reaching *R*^2^ values of 0.801 and 0.755, respectively. This finding highlights a critical physical insight: for biomass estimation, features characterizing the spatial distribution (horizontal and vertical) are more dominant than those characterizing biochemical density.

The advantages of the 3D-DMI framework become evident in multi-sensor combinations. All dual-source models attained high *R*^2^ values exceeding 0.87, significantly outperforming any single-sensor model. Notably, the combination of LiDAR and RGB (*R*^2^ = 0.886) proved highly effective, indicating that the integration of complementary three-dimensional structural information is a primary factor for improving estimation accuracy. Ultimately, the All-Combined model achieved the highest accuracy (*R*^2^ = 0.920), reducing the RMSE to 0.184 kg/m². This suggests that while spatial structural features provide the fundamental basis for biomass estimation, the inclusion of multispectral data introduces essential biochemical dimensions. This integration allows for a more comprehensive representation of the rice canopy, particularly in scenarios where plants may exhibit similar structural volumes but differ in their spectral reflectance characteristics.

The core value of this study lies in establishing a physically interpretable framework that explicitly guides the selection of remote sensing features representing the horizontal, vertical, and density dimensions. By implementing the dual-step feature selection approach, this framework ensures that the input variables possess clear physical correspondences to the structural and physiological traits of the rice canopy. The results confirm that integrating these three physical components is an effective strategy for achieving robust and accurate rice AGB estimation throughout the whole growth period.

### 3.4 Performance of individual features in rice AGB estimation

#### 3.4.1 Evaluation of single-feature predictive capabilities

**Figure 8:**
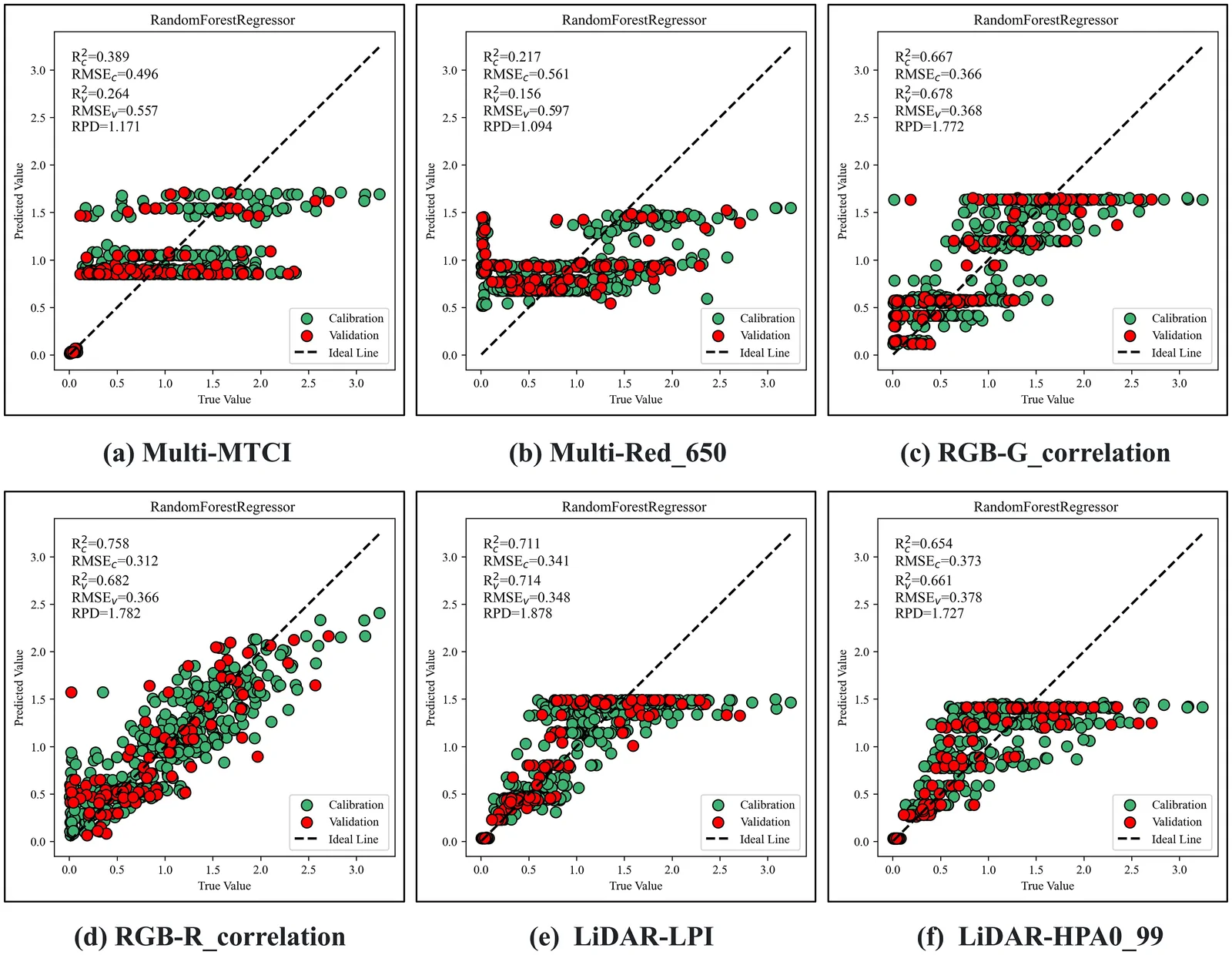
Scatter plots of measured versus predicted rice AGB using single core features. **(a)** Multi-MTCI; **(b)** Multi-Red_650; **(c)** RGB-G_correlation; **(d)** RGB-R_correlation; **(e)** LiDAR-LPI; **(f)** LiDAR-HPA0_99.

To evaluate the capability of individual features in characterizing rice biomass, single-feature regression models were constructed using the RF algorithm. **Table 4** summarizes the predictive performance on the test set, while **Figure 8** visualizes the scatter plots of measured versus predicted values.

**Table 4:** Performance comparison of rice AGB estimation models based on single features using the RF algorithm.

Feature	Physical component	Training set		Test set		RPD
		R^2^_C_	RMSE_C_	R^2^_V_	RMSE_V_	
MTCI	*ρ*	0.389	0.496	0.264	0.557	1.171
Red_650	*ρ*	0.217	0.561	0.156	0.597	1.094
G_correlation	*S*	0.667	0.366	0.678	0.368	1.772
R_correlation	*S*	0.758	0.312	0.682	0.366	1.782
LPI	*S*	0.711	0.341	0.714	0.348	1.878
HPA0_99	*h_d_*	0.654	0.373	0.661	0.378	1.727

The spectral features, MTCI and Red_650, yielded relatively weak predictive constraints when used independently, with test set *R*^2^ values of only 0.264 and 0.156, respectively. This limitation arises because these spectral indices primarily characterize the dry matter density (*ρ*) by responding to leaf pigment content. While effective for monitoring physiological status, single spectral indicators are insufficient to capture the comprehensive volumetric growth information required for accurate AGB estimation, particularly when biomass accumulation is driven by structural expansion rather than pigmentation changes.

In contrast, spatial structural features demonstrated significantly superior accuracy. The RGB texture features, G_correlation and R_correlation, achieved *R*^2^ values of 0.678 and 0.682, respectively. These 2D textures quantify the spatial correlation of gray values, which corresponds to the horizontal arrangement of leaves, thereby reflecting the horizontal projection distribution (*S*). Notably, LiDAR-derived features exhibited the strongest correlation among single sensors. LPI achieved the highest predictive accuracy among all single features (*R*^2^ = 0.714), while HPA0_99 achieved 0.661. This performance is rooted in their physical definitions: LPI characterizes the horizontal canopy occupancy by reflecting structural porosity, while HPA0_99 captures the vertical cumulative distribution ( $h_{d}$). The fact that structural features outperformed spectral features confirms that metrics capturing spatial arrangement are inherently more robust predictors for biomass than those capturing spectral reflectance alone.

#### 3.4.2 Sensitivity and saturation analysis across growth stages

**Figure 9:**
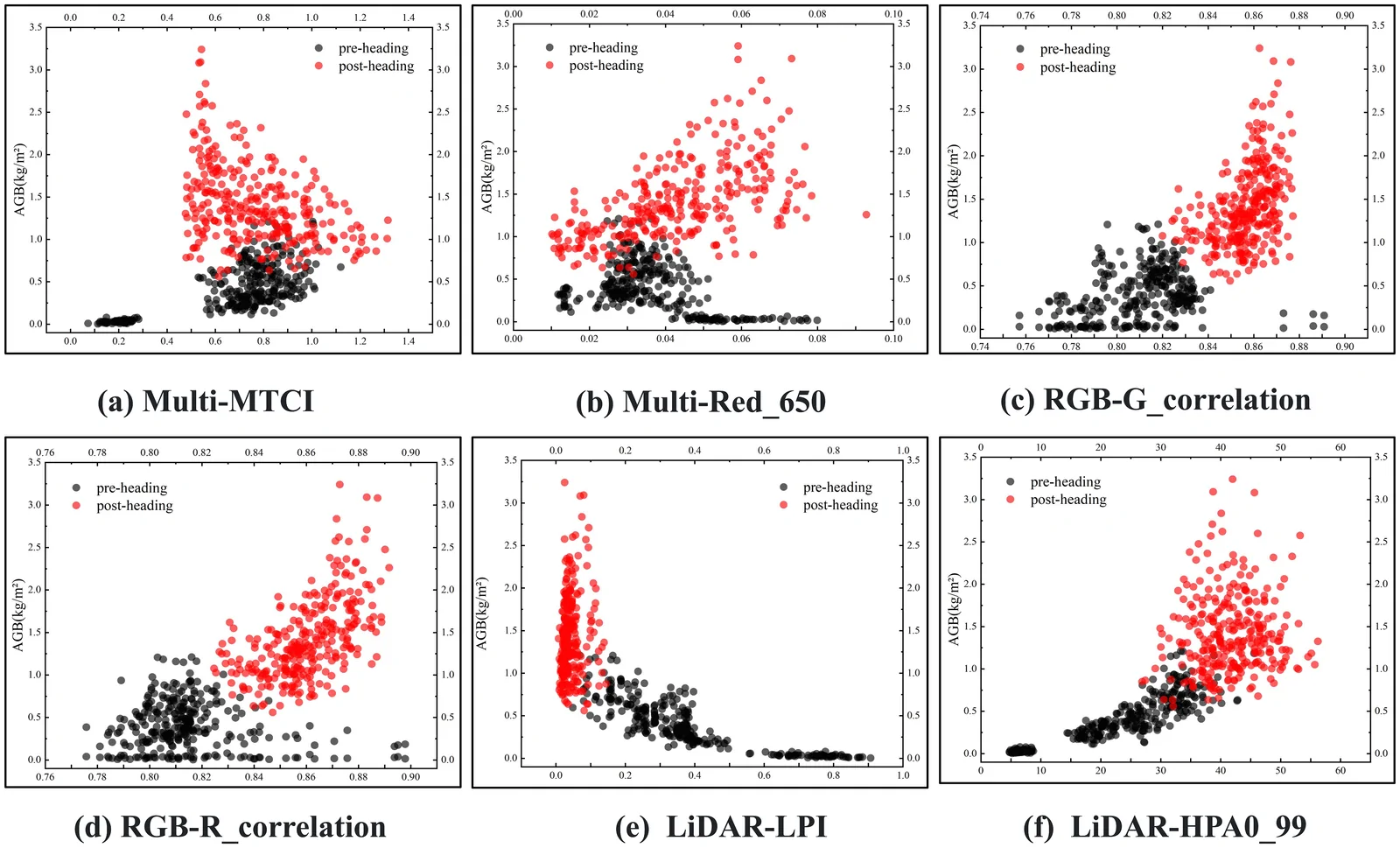
Relationships between individual features and rice aboveground biomass before and after heading. **(a)** Multi-MTCI; **(b)** Multi-Red_650; **(c)** RGB-G_correlation; **(d)** RGB-R_correlation; **(e)** LiDAR-LPI; **(f)** LiDAR-HPA0_99.

To investigate the sensitivity of features across different growth phases, **Figure 9** illustrates the relationship between individual features and AGB segmented by the heading stage. Although the 3D-DMI models based on single features achieved relatively high *R*^2^ values, obvious saturation phenomena occurred in these models, as visualized in the scatter plots of **Figure 8**.

Specifically, nearly horizontal or vertical distributions appeared in the scatter plots for MTCI, Red_650, G_correlation, R_correlation, LPI, and HPA0_99, indicating that these features remained stagnant even as rice AGB continued to change. For the LiDAR-derived LPI feature, for example, **Figure 8E** revealed that the maximum predicted values were approximately 1.498 kg/m², indicating that AGB values exceeding this threshold cannot be accurately predicted by the LPI-based model, thus creating a performance “ceiling.”

Physically, this phenomenon occurred because the LPI during the middle and late growth periods remained nearly constant (see **Figure 9E**). Once the canopy reaches a certain degree of closure, the laser penetration rate stabilizes; however, rice AGB continues to increase due to the continuous accumulation of grain biomass during the grain-filling stage. Similar saturation patterns were observed in other features (**Figures 8**, **9**). In contrast, RGB texture features maintained relatively better linearity in the high-biomass range, explaining why the RGB-based model achieved a slightly higher overall accuracy (*R*^2^ = 0.801) than the LiDAR-based model (*R*^2^ = 0.755). These findings demonstrate that no single feature can independently predict rice AGB across the entire growth period without bias, highlighting the critical importance of the multi-feature fusion approach established in the 3D-DMI framework.

### 3.5 Core feature contribution analysis based on SHAP

**Figure 10:**
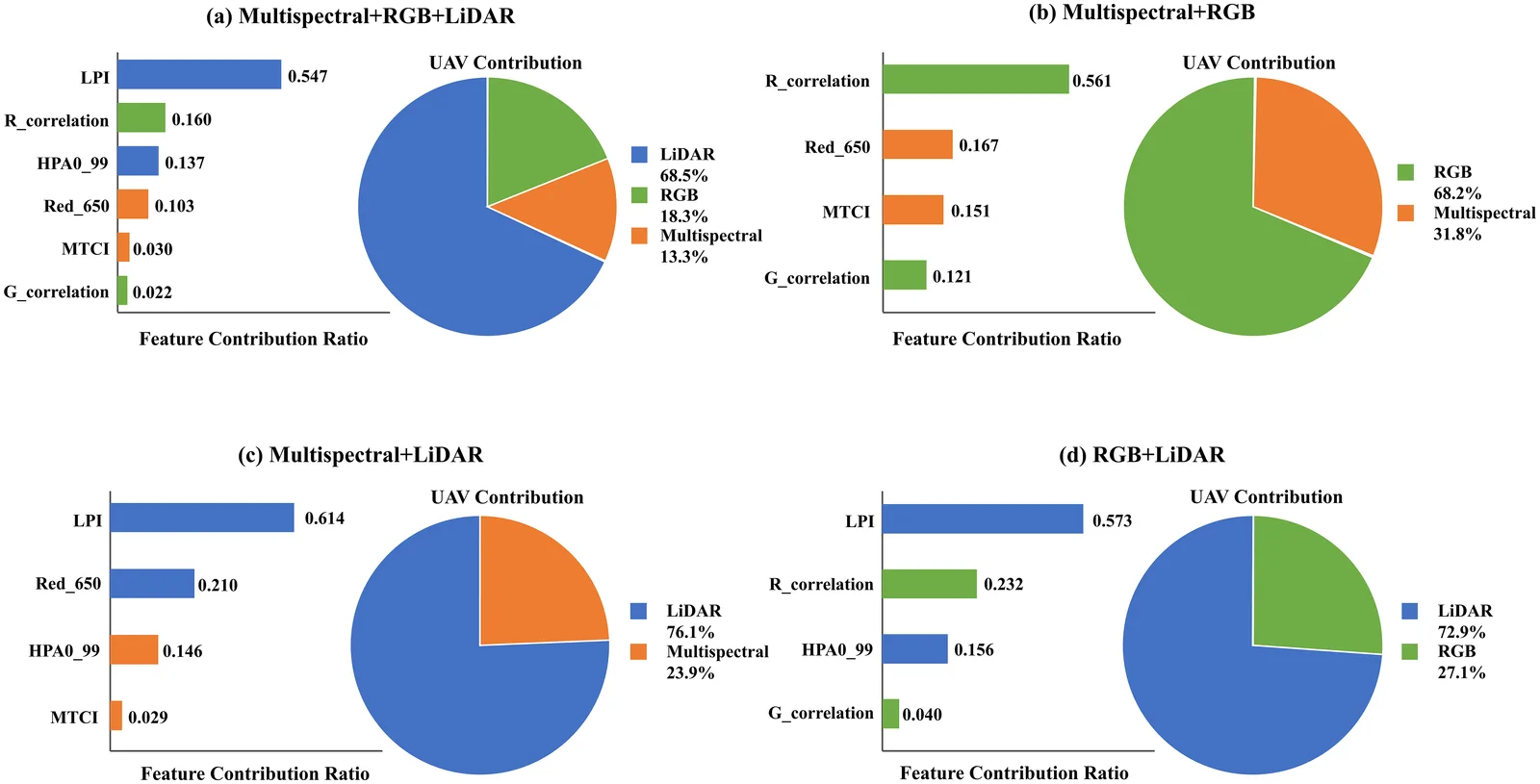
Contribution ratios of core features and sensors to rice AGB estimation under different sensor combinations. **(a)** Multispectral + RGB + LiDAR; **(b)** Multispectral + RGB; **(c)** Multispectral + LiDAR; **(d)** RGB + LiDAR.

To enhance the interpretability of the RF model and quantify the contribution of different sensor features, the SHAP method was employed (Nohara et al., 2022). Specifically, the TreeExplainer, optimized for tree-ensemble models, was utilized to calculate the marginal contribution of each feature to the model output. The global importance was quantified by computing the mean absolute SHAP value across the entire dataset, subsequently normalized to derive the relative contribution ratio. The contribution distributions under different scenarios are visualized in **Figure 10**.

For the 3D-DMI model constructed using the six core features identified through the dual-step feature selection approach (**Figure 10A**), the analysis reveals a clear hierarchy in sensor contribution: LiDAR (68.5%) > RGB (18.3%) > Multispectral (13.3%). LiDAR-derived structural features played the dominant role. LPI, representing the horizontal canopy occupancy, contributed the most at 54.7%, while HPA0_99, characterizing the vertical cumulative distribution, contributed an additional 13.7%. Consequently, the aggregated contribution of the LiDAR-derived structural component reached 68.5%, constituting the primary predictive basis. In comparison, the RGB texture features corresponding to the horizontal projection distribution jointly contributed 18.3%. Meanwhile, the spectral features (Red_650 and MTCI) representing the dry matter density showed the lowest impact, together contributing 13.3%.

In the dual-source combinations (**Figures 10B–D**), contribution ratios adjusted dynamically based on the available information. When structural features were combined with other modalities, the structural component consistently dominated the decision process, reaching up to 76.1%. A critical shift occurred when vertical structural information was excluded: in the Multispectral and RGB scenario, the horizontal arrangement component derived from RGB textures took the primary role, accounting for 68.2% of the contribution. This finding suggests that in the absence of explicit vertical profile information, the model automatically relies on the horizontal spatial arrangement as the primary proxy to infer biomass.

These SHAP results quantify the physical logic of the model and validate the theoretical framework of the 3D-DMI. The dominance of LiDAR-derived information confirms that establishing a volumetric basis is crucial for biomass estimation, as it captures the vertical biomass profile. The stable contribution of RGB features highlights the importance of the horizontal component, which constrains the lateral canopy coverage. Finally, the supplementary role of multispectral features ensures the inclusion of the density component, characterizing internal tissue compactness. The accurate estimation of rice AGB depends on the synergistic interaction of these three physical components, confirming that multi-source fusion is essential for capturing comprehensive growth characteristics.

## 4 Discussion

The core value of this study lies in establishing a physically interpretable three-dimensional dry matter distribution framework for rice AGB estimation. Machine learning is solely utilized as a mathematical tool to solve the implicit expressive relationship between remote sensing variables and AGB. Therefore, to validate the fundamental superiority of the 3D-DMI physical framework over conventional data-driven strategies, we conducted a controlled comparative analysis using the identical dataset. To ensure strict fairness, all three feature selection methods were constrained to output exactly six key features, and the RF algorithm was uniformly employed to train the estimation models. The models were evaluated based entirely on their generalization performance on the independent test set. The performance comparison of these strategies is summarized in **Table 5**.

**Table 5:** Performance comparison of rice AGB estimation using RF algorithm under different feature selection strategies.

No.	Method	Selected feature subset	Test set		RPD
			R^2^_V_	RMSE_V_	
Method 1	RFE-based screening	G_contrast, H30, CHA_50, HPA_30, HPA_95, H90	0.868	0.1794	2.7528
Method 2	Pearson correlation-based screening	LPI, CHA_99, H_max, H_skew, CHA_90, H90	0.7899	0.2807	2.1814
Ours	Proposed 3D-DMI Framework	MTCI, Red_650, G_correlation, R_correlation, LPI, HPA0_99	0.920	0.184	3.544

Method 1, employing the Recursive Feature Elimination (RFE) technique, selected a subset consisting of one RGB feature and five LiDAR structural metrics. This combination achieved a test *R*^2^ of 0.868 and an RPD of 2.7528. The limitation of this strategy is apparent: without a top-down physical guidance, the RFE selection heavily biased towards structural traits while entirely missing the multispectral information. Consequently, it completely failed to characterize the dry matter density, creating an incomplete physical representation of the canopy.

Method 2, driven by Pearson correlation analysis, selected a subset consisting entirely of LiDAR features. This approach resulted in the lowest test performance (*R*^2^ = 0.7899, RMSE = 0.2807). Because LiDAR height metrics generally exhibit high mathematical correlation with AGB, unconstrained statistical algorithms tend to over-select similar structural variables, leading to severe information redundancy. Biologically, this subset completely ignores the horizontal projection distribution and the internal dry matter density, rendering the model unable to accurately estimate AGB during later growth stages when canopy height saturates.

The proposed 3D-DMI framework (Ours) achieved the highest test accuracy (*R*^2^ = 0.920, RMSE = 0.184) and the strongest generalization capability (RPD = 3.544). This success is directly attributed to the explicit physical constraints embedded within the framework. By forcing the selected features to systematically map onto the horizontal projection distribution, the vertical cumulative distribution, and the dry matter density to represent three distinct physical dimensions, the 3D-DMI framework prevents structural redundancy and ensures that the complete 3D physical volume integration process of biomass is precisely captured. This comparative analysis rigorously confirms that integrating a physically meaningful framework is the key to achieving robust and highly accurate rice AGB estimation.

Compared with conventional data-driven feature selection and fusion strategies, the proposed 3D-DMI framework provides a physically constrained organization of multi-source remote sensing variables. In this study, the RFE-based screening method and Pearson correlation-based screening method were used as representative traditional strategies for comparison. The RFE-based method achieved a test *R*^2^of 0.868 but selected mainly structural features, resulting in the absence of multispectral information associated with dry matter density. The Pearson correlation-based method showed lower performance (*R*^2^ = 0.790) because it excessively relied on LiDAR-derived structural variables and ignored the horizontal spatial distribution and internal dry matter density components. In contrast, the proposed 3D-DMI framework achieved a higher accuracy (*R*^2^ = 0.920) by constraining feature selection according to the three physical dimensions of biomass accumulation, including horizontal spatial distribution, vertical spatial structure, and dry matter density.

Furthermore, unlike conventional feature-fusion approaches that directly concatenate multi-source remote sensing variables without considering their biological meanings, the proposed framework establishes explicit relationships between sensor-derived features and biomass components. Compared with previous physically guided biomass estimation approaches, the proposed framework further improves practical applicability by reducing the dependence on field-measured parameters through automatic feature extraction based on MIC and dCor, using machine learning models to capture complex biomass relationships, and introducing SHAP analysis to quantify the contribution of different sensor sources.

Beyond improving estimation accuracy, the three components of the 3D-DMI framework also provide a physiological interpretation of biomass accumulation. These components are not merely mathematical representations but are closely associated with the underlying physiological processes of crop growth. Environmental factors such as nitrogen availability and genetic factors among cultivars can regulate canopy development, plant architecture, and biomass allocation patterns, thereby influencing the characteristics of these three components. Increased nitrogen availability generally promotes leaf expansion, tillering development, and canopy closure, which subsequently affects the horizontal projection distribution and vertical cumulative distribution. Meanwhile, cultivar-specific differences in plant architecture and assimilate partitioning can further modify dry matter density and spatial biomass allocation patterns.

## 5 Conclusions

This study developed a physically interpretable 3D-DMI framework for estimating rice AGB across the entire growth period by fusing multi-source UAV remote sensing data. This framework explicitly characterizes rice biomass through three physical components: dry matter density, horizontal projection distribution, and vertical cumulative distribution. Based on the systematic evaluation of the model, three key findings were revealed.

First, the dual-step feature selection approach based on MIC and dCor effectively identified a core subset of six features that possess clear physical correspondences to the model components. The results demonstrated that features representing spatial structure exhibit stronger physical dependencies with AGB than spectral features. Based on this optimal feature subset, the RF algorithm was proven to be the superior solver (*R*^2^ = 0.920, RMSE = 0.184 kg/m², RPD = 3.544) for reconstructing the complex nonlinear mapping between the 3D-DMI components and rice AGB.

Second, comparative analysis highlighted the necessity of multi-source fusion for full-season estimation. While the RGB-based model (*R*^2^ = 0.801) and LiDAR-based model (*R*^2^ = 0.755) significantly outperformed the multispectral model (*R*^2^ = 0.656), single-sensor approaches were limited by inherent saturation phenomena. Specifically, spectral indices and the LiDAR-derived LPI reached performance ceilings during high-biomass stages, whereas RGB textures maintained relatively better linearity. This confirms that spatial distribution features (horizontal and vertical) are more critical than biochemical density features for biomass characterization, yet no single sensor can independently capture the complete growth trajectory without bias.

Third, the comprehensive 3D-DMI fusion model achieved the highest accuracy and robustness. SHAP-based analysis quantified the hierarchical contribution of the components, where LiDAR-derived structural features played the dominant role (68.5%), followed by RGB texture features (18.3%) and multispectral features (13.3%). This confirms that three-dimensional structural information constitutes the fundamental basis for AGB estimation. Despite their lower contribution ratios, multispectral and RGB features provide essential refinements: the horizontal projection distribution constrains the lateral arrangement, while the dry matter density accounts for internal physiological densification. These findings validate the 3D-DMI framework, demonstrating that the synergistic interaction of horizontal, vertical, and density dimensions ensures high-precision rice phenotyping.

Beyond its methodological contribution, the proposed framework provides a physically interpretable and non-destructive tool for precision agriculture, with potential applications in crop growth monitoring, nitrogen management, yield prediction, and breeding-related phenotypic assessment.

Despite the promising performance of the proposed 3D-DMI framework, several limitations should be acknowledged. First, the current validation dataset was primarily collected from a single experimental site and one growing season, which may limit the evaluation of model generalization under diverse environmental conditions. Second, although the framework effectively integrates multi-source UAV observations for rice AGB estimation, its applicability to other crops with different canopy architectures requires further investigation. Future studies should incorporate multi-year, multi-location, and multi-cultivar datasets to further validate and improve the robustness, transferability, and practical applicability of the proposed framework.

## Data Availability

The raw data supporting the conclusions of this article will be made available by the authors, without undue reservation.
